# Health research improves healthcare: now we have the evidence and the chance to help the WHO spread such benefits globally

**DOI:** 10.1186/s12961-015-0006-y

**Published:** 2015-03-03

**Authors:** Stephen R Hanney, Miguel A González-Block

**Affiliations:** Health Economics Research Group, Brunel University London, Kingston Lane, Uxbridge, UB8 3PH UK; Universidad Anáhuac, Av. Universidad Anáhuac 46, Lomas Anáhuac, 52786 Huixquilucan Mexico City, Mexico

**Keywords:** Assessing research benefits, Capacity building, Diseases of poorest countries, Global health, Global Observatory, Platforms for research implementation, Research expenditure, Screening for abdominal aortic aneurysms, World Health Organization, World Health Report 2013

## Abstract

There has been a dramatic increase in the body of evidence demonstrating the benefits that come from health research. In 2014, the funding bodies for higher education in the UK conducted an assessment of research using an approach termed the Research Excellence Framework (REF). As one element of the REF, universities and medical schools in the UK submitted 1,621 case studies claiming to show the impact of their health and other life sciences research conducted over the last 20 years. The recently published results show many case studies were judged positively as providing examples of the wide range and extensive nature of the benefits from such research, including the development of new treatments and screening programmes that resulted in considerable reductions in mortality and morbidity.

Analysis of specific case studies yet again illustrates the international dimension of progress in health research; however, as has also long been argued, not all populations fully share the benefits. In recognition of this, in May 2013 the World Health Assembly requested the World Health Organization (WHO) to establish a Global Observatory on Health Research and Development (R&D) as part of a strategic work-plan to promote innovation, build capacity, improve access, and mobilise resources to address diseases that disproportionately affect the world’s poorest countries.

As editors of *Health Research Policy and Systems* (*HARPS*), we are delighted that our journal has been invited to help inform the establishment of the WHO Global Observatory through a Call for Papers covering a range of topics relevant to the Observatory, including topics on which *HARPS* has published articles over the last few months, such as approaches to assessing research results, measuring expenditure data with a focus on R&D, and landscape analyses of platforms for implementing R&D. Topics related to research capacity building may also be considered. The task of establishing a Global Observatory on Health R&D to achieve the specified objectives will not be easy; nevertheless, this Call for Papers is well timed – it comes just at the point where the evidence of the benefits from health research has been considerably strengthened.

## Editorial

The start of 2015 sees a dramatic increase in the body of evidence demonstrating the benefits arising from health research. Throughout 2014, the higher education funding bodies in the UK conducted an assessment of research, termed the Research Excellence Framework (REF), in which, for the first time, account was taken of the impact on society of the research undertaken. As part of this, UK universities and medical schools produced 1,621 case studies that aimed to show the benefits, such as improved healthcare, arising from examples of their health and other life sciences research conducted over the last 20 years. Panels of experts, including leading academics from many countries, published their assessments of these case studies in December 2014 [1], with the full case studies and an analysis of the results being made public in January 2015 [2,3].

As we recently anticipated [4], the expert panels concluded that the case studies did indeed overwhelmingly illustrate the wide range and extensive nature of the benefits from health research. Main Panel A covered the range of life sciences and its overview report states: “*MPA* [Main Panel A] *believes that the collection of impact case studies provide a unique and powerful illustration of the outstanding contribution that research in the fields covered by this panel is making to health, wellbeing, wealth creation and society within and beyond the UK*” [3], p. 1. The section of the report covering public health and health services research also notes that: “*Outstanding examples included cases focused on national screening programmes for the selection and early diagnosis of conditions*” [3], p. 30. In their section of the report, the international experts say of the REF2014: “*It is the boldest, largest, and most comprehensive exercise of its kind of any country’s assessment of its science*” [3], p. 20.

The REF2014 is therefore attracting wide international attention. Indeed, some of the methods used are already informing studies in other countries, including, for example, an innovative assessment recently published in *Health Research Policy and Systems* (*HARPS*) identifying the beneficial effects made on healthcare policies and practice in Australia by intervention studies funded by the National Health and Medical Research Council [5].

The REF also illustrates that, even when focusing on the research from one country, there are examples of studies in which there has been international collaboration and which have built on research conducted elsewhere. For example, one REF case study on screening describes how a major UK randomised controlled trial of screening for abdominal aortic aneurysms (AAA) involving 67,800 men [6,7] was the most significant trial globally. The trial provided the main evidence for the policy to introduce national screening programmes for AAA for men reaching 65 throughout the UK [2]. The importance of this trial lay partly in its size, given that it accounted for over 50% of the men included in the meta-analyses performed in the 2007 Cochrane review [8] and the 2009 practice guideline from the US Society for Vascular Surgery [9]. Nevertheless, two of the three smaller studies that were also included in these two meta-analyses came from outside the UK, specifically from Denmark [10] and Australia [11].

Moreover, a recent paper published in *HARPS* also included descriptions of how the research contributing to new interventions often comes from more than one country. These accounts are included in a separate set of seven extensive case studies constructed to illustrate innovative ways to measure the time that can elapse between research being conducted and its translation into improved health [12]. While being a separate set of case studies, one of them does, nevertheless, explore the international timelines involved in research on screening for AAA, and, in addition to highlighting the key role of the UK research, it also highlights that the pioneering first screening study using ultrasound had been conducted in 1983 on 73 patients in a US Army medical base [13].

These case studies therefore further reinforce the well-established argument that health research progress often involves contributions from various countries. However, as has long been argued, not all populations fully share the benefits. In recognition of this, in May 2013, the World Health Assembly requested the World Health Organization (WHO), in its resolution 66.22, to establish a Global Observatory on Health Research and Development as part of a strategic work-plan to promote innovation, build capacity, improve access, and mobilise resources to address diseases that disproportionately affect the world’s poorest countries [14].

As editors of *HARPS*, we are delighted that our journal has been invited to help inform the establishment of the WHO Global Observatory by publishing a series of papers whose publication costs will be funded by the WHO. In support of this WHO initiative, Taghreed Adam, John-Arne Røttingen, and Marie-Paule Kieny recently published a Call for Papers for this series [15], which can be accessed through the *HARPS* webpage.

The aim of the series is “*to contribute state-of-the-art knowledge and innovative approaches to analyse, interpret, and report on health R&D information…* [and] *to serve as a key resource to inform the future WHO-convened coordination mechanism, which will be utilized to generate evidence-informed priorities for new R&D investments to be financed through a proposed new global financing and coordination mechanism for health R&D*” [15], p. 1. The Call for Papers covers a range of topics relevant to the aims of the Global Observatory. These include ones on which HARPS has published articles in the last few months, such as approaches to assessing research results, as seen in the Australian article described above [5]; papers measuring expenditure data with a focus on R&D, as described in a recent Commentary by Young et al. [16]; and landscape analyses of platforms for implementing R&D, as described in the article by Ongolo-Zogo et al. [17], analysing knowledge translation platforms in Cameroon and Uganda, and partially in the article by Yazdizadeh et al. [18], relaying lessons learnt from knowledge networks in Iran.

Adam et al. also make clear that the topics listed in the Call for Papers are examples and that the series editors are also willing to consider other areas [15]. Indeed, in the Introduction to the Call for Papers, the importance of capacity building is highlighted. This, too, is a topic described in recent papers in *HARPS*, such as those by Ager and Zarowsky [19], analysing the experiences of the *Health Research Capacity Strengthening* initiative’s Global Learning program of work across sub-Saharan Africa, and by Hunter et al. [20], describing needs assessment to strengthen capacity in water and sanitation research in Africa.

Finally, as we noted in our earlier editorial [4], the *World Health Report 2013: Health Research for Universal Coverage* showed how the demonstration of the benefits from health research could be a strong motivation for further funding of such research. As the Report states, “*adding impetus to do more research is a growing body of evidence on the returns on investments … there is mounting quantitative proof of the benefits of research to health, society and the economy*” [21]. We noted, too, that since the Report’s publication in 2013, there had been further examples from many countries of the benefits from medical research. The REF2014 in the UK signifies an additional major boost to the evidence that a wide range of health research does contribute to improved health and other social benefits. The results of such evaluations highlight the appropriateness of the WHO’s actions in attempting to ensure all populations share the benefits of health research endeavours by creating the Global Observatory on Health Research and Development. This will not be an easy task, but we welcome the opportunity afforded by the current Call for Papers for researchers and other stakeholders to engage with this process and influence it [15].

## Abbreviations

AAA: Abdominal aortic aneurysms
HARPS: Health Research Policy and Systems
MPA: Main Panel A
R&D: Research and development
REF: Research Excellence Framework
WHO: World Health Organization
